# Therapeutic role of hyperinsulinemia/euglycemia in aluminum phosphide poisoning

**DOI:** 10.1097/MD.0000000000004349

**Published:** 2016-08-07

**Authors:** Hossein Hassanian-Moghaddam, Nasim Zamani

**Affiliations:** a Toxicological Research Center, Department of Clinical Toxicology, Loghman-Hakim Hospital, Shahid Beheshti University of Medical Sciences; b Excellence Center of Clinical Toxicology, Iranian Ministry of Health, Tehran, Iran.

**Keywords:** aluminum phosphide, GIK protocol, insulin, poisoning, treatment

## Abstract

**Background:**

Different protocols have been suggested to treat aluminum phosphide (ALP) poisoning. We aimed to evaluate the possible therapeutic effect of hyperinsulinemia/euglycemia (HIE) in treatment of ALP poisoning.

**Methods:**

In a prospective interventional study, a total of 88 ALP-poisoned patients were included and assigned into HIE group undergoing glucose/insulin/potassium (GIK) protocol and a control group that was managed by routine conventional treatments. The 2 groups were then compared regarding the signs and symptoms of toxicity and their progression, development of complications, and final outcome to detect the possible effect of GIK protocol on the patients’ course of toxicity and outcome.

**Results:**

The 2 groups were similar in terms of demographic characteristics and on-arrival vital signs and lab tests. Using GIK protocol resulted in significantly longer hospital stays (24 vs 60 hours; *P* < 0.001) and better outcomes (72.7% vs 50% mortality; *P* = 0.03). Regression analysis showed that GIK duration was an independent variable that could prognosticate mortality (odds ratio [95% confidence interval] = 1.045 [1.004,1.087]). The risk of mortality decreased by 4.5% each hour after initiation of GIK.

**Conclusion:**

GIK protocol improves the outcome of ALP poisoning and increases the length of hospital stay.

## 1 Introduction

Annually, almost 300,000 deaths occur worldwide due to pesticide poisoning, probably some of whom die due to aluminum phosphide (ALP) intoxication. ALP was first introduced as a pesticide in India.^[1,2]^ Its tablets are commonly used in Iran (especially in the northern parts) due to their low cost, high efficacy, and availability causing an increased risk of intentional and accidental poisonings and death. ALP poisoning was reported in only 59 cases between 1900 and 1958 with 26 of the poisoned patients dead.^[3,4]^ This rate has dramatically increased within the recent 35 years due to its significant ingestion to commit suicide. It is one of the most common causes of death due to poisoning in Iran with an increased incidence of fatal ALP poisoning from 5.2 to 37 per million of population in 2006 and 2013 in Tehran.^[5–7]^

ALP poisoning has no specific antidote, and therefore, different modalities have been suggested for its treatment, to date. Patients generally die due to multi-organ failure. From the factors associating with poor prognosis, cardiocirculatory collapse and metabolic acidosis are probably the most important ones that do not respond to conventional therapies.^[8]^ Animal and human studies showed that high exogenous insulin had strong positive inotropic effects.^[9–11]^ Hyperinsulinemia–euglycemia (HIE)—by administrating glucose, insulin, and potassium (GIK)—assists in myocardial uptake of carbohydrates which are the preferred fuel substrate of the heart under stressed conditions.^[12]^ The putative mechanism is correction of calcium antagonist or beta-blockers-induced hypoinsulinemia, leading to improved cell carbohydrate metabolism, increased myocardial contractility and peripheral vascular resistance, and correction of acidosis. The treatment is not expected to improve conduction block or bradycardia. HIE was first introduced as a possible treatment of ALP poisoning in 2008.^[13]^ Although this treatment was tested on only 5 patients at the time of publication of the preliminary results with it and is currently recommended as a treatment in ALP poisoning,^[14]^ no study has proved its efficacy even retrospectively.

### 1.1 Importance

Although mortality of ALP poisoning has been reported to be as high as 90% in some studies, no antidote is available to treat the patients.

### 1.2 Goals of this investigation

The aim of the present study is to evaluate the probable therapeutic effect of GIK protocol in treatment of ALP poisoning.

## 2 Methods

### 2.1 Study design

A prospective interventional study was conducted using a convenient sampling of all patients with ALP ingestion. Further confirmation of ALP exposure was done by silver nitrate test (SNT) or development of clinical signs and symptoms of toxicity. Participants entered into either the intervention group (GIK protocol + conventional standard therapy) or control group (conventional standard treatment only). Conventional standard therapy included using inotropes, fluids and electrolytes resuscitation, intubation, mechanical ventilation, and antiarrhythmic agents if indicated.

### 2.2 Setting

Loghman-Hakim Hospital Poison Center is the main referral hospital for poisoned patients in Tehran (the capital city with 12.5 million permanent and 6.5 million temporary residents). Over 24,000 to 27,000 intoxicated patients refer to this center annually, of whom, almost 10,000 to 12,000 are hospitalized. There are almost 230 to 280 ALP suspected cases annually.^[4,5]^

### 2.3 Sample size

Considering an average mortality of 70% in aluminum phosphide poisoning,^[14]^ the desired 95% level of confidence, the desired ± 10% precision of the estimate, a sample size of 81 was achieved to study the effect of new intervention in reducing mortality. Forty-four subjects per group were recruited after consideration of dropouts.

### 2.4 Randomization

**Figure 1:**
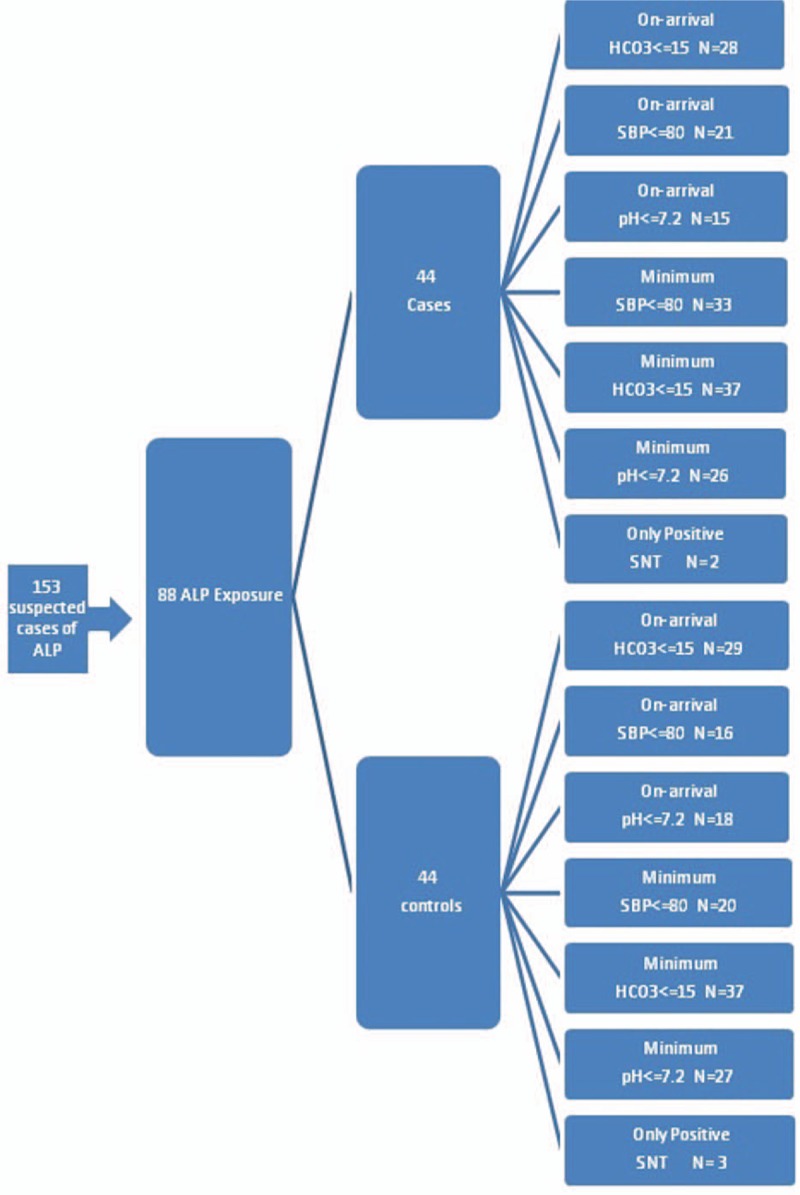
Patients allocation into intervention/control groups.

Between 2006 and 2012, sampling was conveniently performed. Whenever the authors were on shift and available at hospital and confirmed ALP poisoning in referred cases, the senior attending physician (first author) visited the patients and entered them into intervention group. There was no gender preference for the allocation. When other attending physicians of the department were on shift, patients were included in the conventional therapy group (control group). Written consent forms were taken from the patients or their next of kin if they were included in the intervention group. All relevant information pack was provided to next of kin and the patients. Patients with a positive history of ALP exposure with either a positive SNT result or a systolic blood pressure (SBP) of <80 mm Hg/a serum bicarbonate level of 15 meq/L or less/a pH of 7.2 or less were considered to be ALP-poisoned and included (Fig. 1). Actually, SNT was done in all patients but since it is a relatively low-sensitive test and may be falsely negative in some patients, the patients were also considered to be ALP-poisoned if they gave the history of ALP ingestion and developed hypotension or had abnormal pH and bicarbonate levels even with a negative SNT. Even if the patients had no such manifestations on presentation but developed them during their hospitalization period, they were included. They were otherwise excluded. Also, if the patients died within 2 hours of admission (whether in the intervention or in the control group), they were excluded. This was mainly due to the fact that we thought the patients in the intervention group needed up to 2 hours to show the effects of GIK protocol.

### 2.5 Interventions

**Figure 2:**
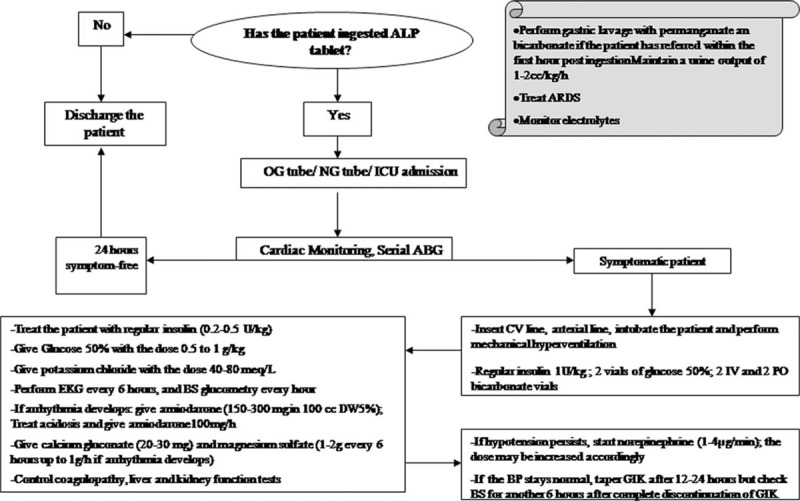
Algorithm used for patient management during the study.

The study was approved by the local ethics committee of Shahid Beheshti University of Medical Sciences. Conservative treatments included intubation and mechanical ventilation, intravenous fluid therapy (with crystalloids), antiarrhythmic medications, calcium gluconate, magnesium sulfate, bicarbonate, and inotropes. The intervention group was treated on the basis of the suggested protocol for study conduction (Fig. 2), while the controls were only given conventional treatments. The loading dose of regular insulin was set at 1 IU/kg followed by 0.2 to 0.5 U/kg/h. Glucose was given at the dose to maintain the serum glucose at around 150 mg/dL. Blood glucose and serum potassium were checked every hour and every 4 hours, respectively. Response to the protocol was defined as increase of SBP to higher than 90 mm Hg or correction of acidosis. If the patients did not respond to this dose of insulin, the dose was increased to a maximum of 3 IU/kg/h. Additional potassium was given to maintain serum potassium at 3.5 to 4.5 meq/L. If GIK protocol was initiated, it was continued for at least 12 to 24 hours. In case the patient was stable, and inotropes were discontinued without later re-evolution of the clinical manifestations, a try was made to taper insulin dose; if not, the patient was maintained on the protocol until complete stability of the vital signs and complete discontinuation of the inotropes. For tapering, the insulin dose was reduced to 1/2 to 2/3 of the initial dose in each visit. Between the visits (usually every 8 hours), the patients were evaluated for possible return of their hypotension and acidosis. If their signs returned, the dose was again increased; if not, tapering was continued until complete discontinuations of insulin. Hypoventilation was defined as patients’ arterial blood gas analyses PCO_2_ levels higher than the expected levels with the Henderson–Hasselbalch equation.^[15]^ Bradycardia was defined as pulse rates less than 60 beats per minute.

### 2.6 Data collection and processing

Patients’ data including age, sex, on-arrival vital signs and lab tests, minimum systolic and diastolic blood pressures (SBP and DBPs) during the course of treatment until discharge or death, signs and symptoms on admission, treatments given, hospital stay, and the final outcome were recorded and entered into social package for statistical sciences software version 17.

### 2.7 Outcome measures

Outcome measures were patients’ mortality and their length of stay.

### 2.8 Primary data analysis

Kolmogorov–Smirnov test was used to determine normal and non-normal distributions of the variables, for whose description, mean (±SD) and median (interquartile range) were given. For qualitative variables, percent of frequency was provided. To compare normally distributed continuous variables between GIK and conventional standard therapy groups, *t* test was used. Mann–Whitney *U* test was performed to compare differences between 2 independent groups when the dependent variable was continuous, but not normally distributed. For the assessment of association between categorical variables, a chi-square test was applied. Pearson correlation coefficient was used for assessing the severity of association between continuous variables. Logistic model determined independent variables predicting death in these patients. Odds ratio (OR) and 95% confidence interval (CI) were used for expressing the severity of this association. A *P* value less than 0.05 was considered to be statistically significant.

## 3 Results

From a total of 153 cases referred with possible ALP ingestion, 67 were excluded due to unavailable or negative SNT results as well as lack of development of clinical manifestations. These cases might have ingested nontoxic tablets or might actually be mild ALP-poisoned whose diagnoses could not be confirmed (Fig. 1). A total of 88 patients were included and assigned into 2 44-patient groups. Forty-three were male (49%). Mean age of the patients was 26.2 ± 8.5 years, and median time elapsed between ingestion and presentation was 3 [Inter Quartile Range (IQR): 2,6] (range: 0.5–24) hours. The patients’ demographic characteristics, on-arrival vital signs, lab tests, and Glasgow coma scale (GCS) are shown in Table 1. As depicted, the 2 groups were similar regarding all the abovementioned variables.

**Table 1:**
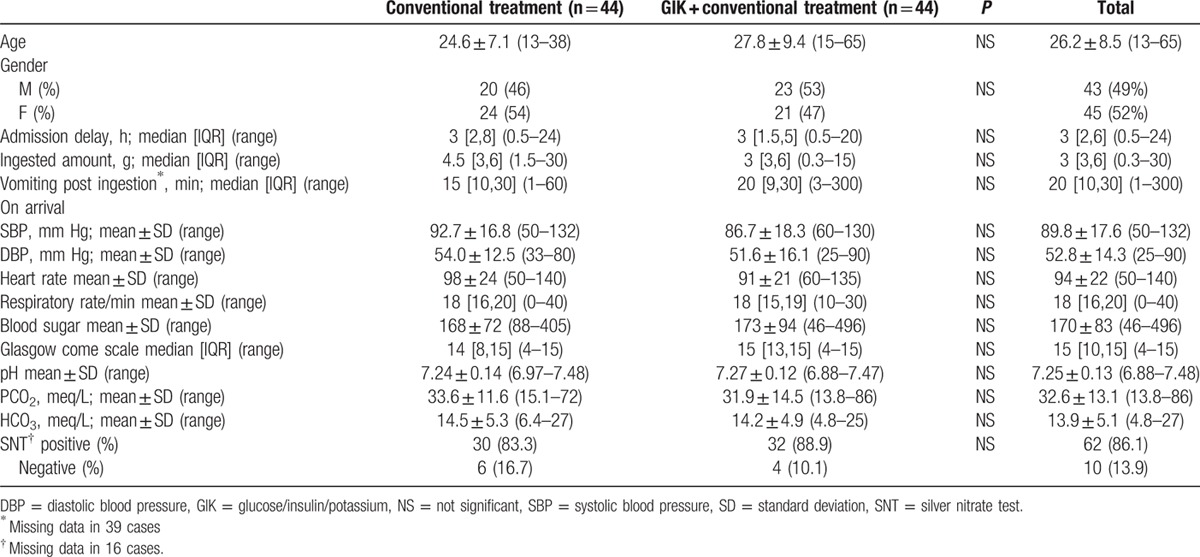
On-arrival characteristics of interventions and controls (n = 88).

After treatment by standard conventional versus GIK protocols, it was revealed that mean SBP (81.1 ± 19.9 vs 73.8 ± 13.5 mm Hg), hospital stay (24 vs 60 hours), and death (72.7% vs 50%) significantly differed between the 2 groups. Although the patients in the standard conventional group had higher blood pressures and seemed more stable regarding their vital signs, they survived less hours and died more (*P* values were <0.001 and 0.03, respectively; Table 2).

**Table 2:**
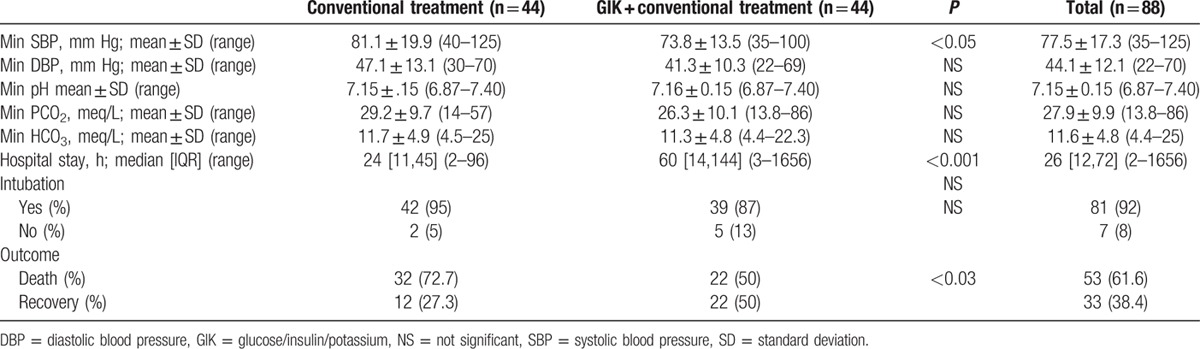
Late characteristics of interventions and controls (n = 88).

**Figure 3:**
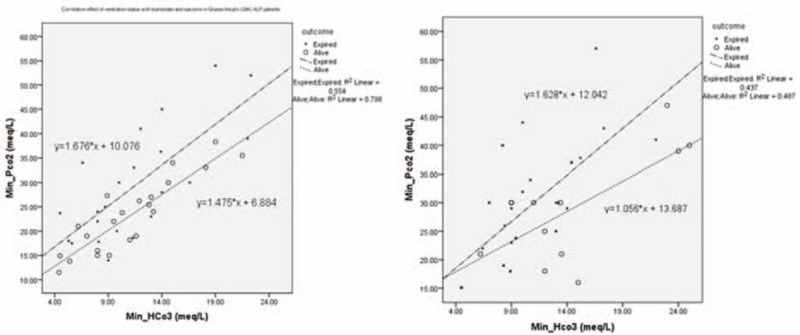
Correlation between ventilation status and bicarbonate/outcome in glucose/insulin/potassium (left) and conventional (right) groups.

When comparing the survivors and nonsurvivors of the 2 groups (Table 3), we found that in the intervention group, on-arrival and minimum pH, hypoventilation, SBP, and on-arrival bicarbonate, blood sugar, and GCS differed significantly between those who died and those who survived. In the control group, on-arrival and minimum pH, hypoventilation, on-arrival GCS, minimum PCO_2_, protocol duration, and insulin cumulative dose were significantly different between the survivors and nonsurvivors (*P* < 0.001). It should be mentioned that median [IQR] (range) of insulin dose/h, duration of GIK administration, and cumulative dose of insulin were 35 [30,60] (6–94), 15 [6,38] (2–134), and 525 [240,2160] (40–11,658) in the GIK group, respectively. Figure 3 shows the correlation between ventilation status and bicarbonate/outcome in GIK and standard conventional groups. Failure to hyperventilate in response to acidosis was a poor prognostic factor (*P* < 0.001).

**Table 3:**
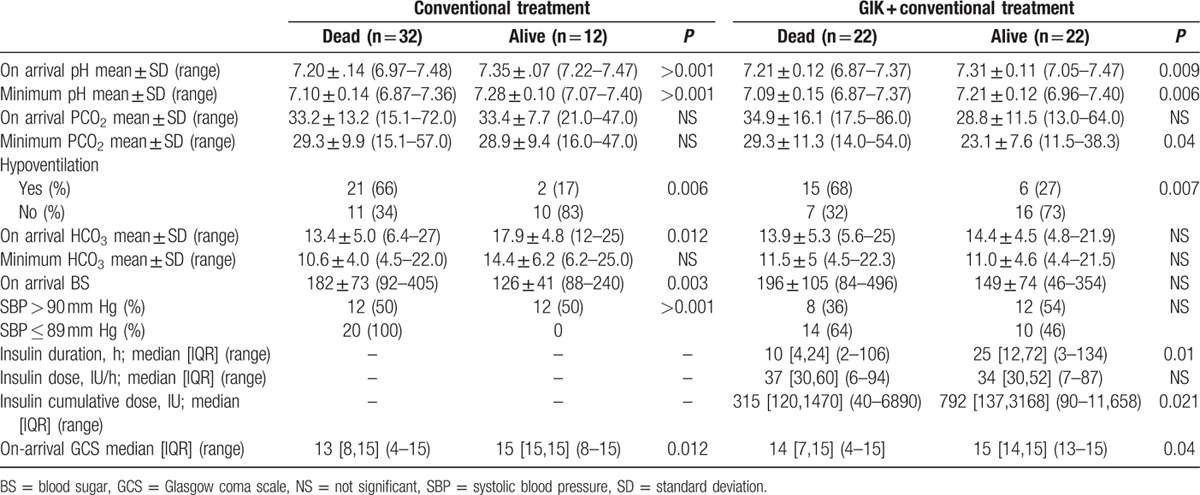
Comparison of on arrival blood gas and later hyperventilation effect and selected characteristics on outcome.

All 8 patients with bradycardia died (*P* < 0.02). Regression analysis showed that GIK duration was an independent variable that could prognosticate mortality (OR [95% CI] = 1.045 [1.004,1.087]); model significance and Nagelkerke *R* square of 0.032, 0.610 showed that risk of mortality decreased by 4.5% each hour after initiation of GIK.

## 4 Discussion

Use of HIE and GIK protocol was first advocated in treatment of toxicity by beta-blockers and calcium channel blockers (CCB) in clinical toxicology.^[16]^ Hypoinsulinemia appears to be a critical factor in CCB overdose. Myocytes oxidize free fatty acids for energy while in shock state (such as ALP poisoning), they switch to glucose utilization. Hypoinsulinemia may prevent glucose uptake by myocytes ensuingloss of inotropy and decreased peripheral vascular resistance. This may explain the fact that hyperglycemia is a poor prognostic factor in ALP poisoning as confirmed by our results and shown by previous studies.^[17]^ As tissue perfusion falls, decreased delivery of glucose deprives myocytes of needed fuel. Continuation of this cycle leads to hemodynamic deterioration, shock, and death.^[18,19]^

The exact mechanism of action of HIE therapy is poorly defined. It improves inotropy and peripheral vascular resistance and reverses acidosis by improving myocyte carbohydrate uptake and utilization. In addition, this therapy may promote the metabolism of lactate and limit metabolic acidosis common in ALP poisoning.^[18]^ Engebretsen et al^[18]^ described that efficacy of HIE was due to increased inotropy and increased intracellular glucose transport. They mentioned different high-dose insulin treatment protocols. When first introduced, insulin doses were cautiously initiated at 0.5 IU/kg bolus dose followed by a 0.5 to 1 IU/kg/h continuous infusion. With increasing clinical experience and publication of animal studies, high-dose insulin was recommended. Doses up to 1 IU/kg of bolus insulin followed by doses as high as 1 to 10 IU/kg/h of continuous infusion were even advocated. Although the optimal regimen is still to be determined, bolus doses up to 10 IU/kg and continuous infusions as high as 22 IU/kg/h have been administered with good outcomes and minimal adverse events.^[19]^

The possible therapeutic effect of GIK protocol on ALP-poisoned patients was first suggested in 2008 when Hassanian-Moghaddam^[13]^ showed promising results with this protocol in 5 cases of ALP poisoning. Although this treatment is currently advocated as a routine treatment in ALP-poisoned patients in our center, no study has evaluated its efficacy in these patients.

As the results show, GIK protocol can significantly increase the hospital stay and improve the final outcome of the patient. Insulin—as a vasodilator—may also explain the difference of minimal SBP between the groups.^[20,21]^ Even in the survivors of both groups, minimum SBP was less in the patients of the GIK group (*P* = 0.04). The other interesting findings were the probable protective effect of hyperventilation and tachycardia. The more the patients hyperventilated, the more they survived, and all patients with bradycardia died. These findings emphasize the possible role of hyperventilation in treatment of ALP-poisoned patients; meanwhile, higher PCO_2_ levels and bradycardia may be used as poor prognostic factors in this toxicity.

Type of patient selection (nonblinded randomization) is definitely a limitation of the present study. We only entered the patients who referred during our own shifts when we could be available for at least the first hours of initiation of GIK protocol. The nature of shock was not well established as the patients were not investigated by echocardiography or Swan Ganz catheter or even lactometer. Also, we did not document the possible complications of our treatments especially insulin. Exclusion of early deaths (those within the first 2 hours after admission) is definitely another limitation of our study which was inevitable because GIK sometimes takes several hours to affect.^[12]^ Other clinically relevant indices such as frequency or duration of mechanical ventilation and time or total dose of vasoactive support were not evaluated, either. Prospective randomized controlled trials are warranted to evaluate the efficacy of this treatment, although it seems that depriving the patients from this treatment seems unethical.

Other advanced novel techniques like extracorporeal life support may save the patients and GIK protocol may increase its success rate.^[22]^

## 5 Conclusion

Insulin probably improves inotropy by altering cell metabolism from fatty acids to carbohydrates and restoring calcium flux resulting in improvement of cardiac contractility. In ALP poisoning, GIK protocol stabilizes cardiovascular system, increases hospital stay, and improves the survival rate.
